# Separation and Purification of Aflatoxins by Centrifugal Partition Chromatography

**DOI:** 10.3390/toxins11060309

**Published:** 2019-05-30

**Authors:** Gábor Endre, Zsófia Hegedüs, Adiyadolgor Turbat, Biljana Škrbić, Csaba Vágvölgyi, András Szekeres

**Affiliations:** 1 Departement of Microbiology, Faculty of Science and Informatics, University of Szeged, Közép fasor 52, H-6726 Szeged, Hungary; egabcy@gmail.com (G.E.); hegedus.zsofia95@gmail.com (Z.H.); adiyadolgor_turbat@yahoo.com (A.T.); mucor1959@gmail.com (C.V.); 2 Doctoral School in Biology, Faculty of Science and Informatics, University of Szeged, H-6720 Szeged, Hungary; 3 Faculty of Technology, University of Novi Sad, Bulevar cara Lazara 1, 21000 Novi Sad, Serbia; biljana@tf.uns.ac.rs

**Keywords:** aflatoxin purification, centrifugal partition chromatography, ternary system, separation

## Abstract

Aflatoxins are mycotoxins that are produced by several species of filamentous fungi. In the European Union, the concentration limits for this group of mycotoxins in food and feed products are very low (on the order of parts per billion). Thus, relatively high amounts of these substances in their pure forms are required as reference standards. Chromatographic techniques based on solid stationary phases are generally used to purify these molecules; however, liquid–liquid chromatographic separations may be a promising alternative. Therefore, this study proposes a liquid–liquid chromatographic method for the separation of four aflatoxins and impurities. To optimise the method, numerous biphasic solvent systems (chloroform-, acetone- and acetic acid-based systems) were tested and evaluated in terms of their effectiveness at partitioning aflatoxins; the toluene/acetic acid/water (30:24:50, v/v/v/%) system was found to be the most efficient for application in centrifugal partition chromatographic instrument. Using liquid–liquid instrumental separation, the four aflatoxins, namely B_1_ (400 mg), B_2_ (34 mg), G_1_ (817 mg) and G_2_ (100 mg), were successfully isolated with 96.3%–98.2% purity from 4.5 L of *Aspergillus parasiticus* fermented material in a 250 mL centrifugal partition chromatography column. The identities and purities of the purified components were confirmed, and the performance parameters of each separation step and the whole procedure was determined. The developed method could be effectively used to purify aflatoxins for analytical applications.

## 1. Introduction

**Figure 1:**
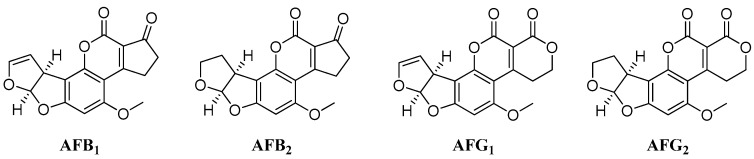
Structures of the four main aflatoxins.

Aflatoxins (AFs) are mycotoxins produced as secondary metabolites mainly by two Aspergillus species, namely *A. parasiticus* and *A. flavus*. Four main AFs, B_1_, G_1_, B_2_ and G_2_ exist (Figure 1), all of which are toxic to both humans and animals [1,2,3]. Consuming food contaminated with AFs could lead to serious health problems, including acute hepatic necrosis, acute liver failure and liver cancer [4,5]. Among these compounds, AFB_1_ is the most toxic; it is associated with teratogenic, mutagenic and carcinogenic effects [6] and may cause lesions mainly in the liver [7] but also in other exposed organs systems [8]. Because of the dangerous nature of AFs, very low limits (on the order of parts per billion) for these compounds in various food products and commodities are set in the European Union and around the world; the maximum residual limits for total AF contamination range from 4 to 10 µg/kg [9].

Numerous methods for determining AF concentrations using various matrices have been proposed [10,11,12]. However, high performance liquid chromatography coupled with and ultraviolet detector (HPLC-UV) has also been used to determine AF concentrations in food products [13], the most popular technique is based on the use of a fluorescence detector regarding the existed fluorophores and additional derivatizations [14,15,16,17]. Furthermore, HPLC can be coupled with mass spectrometry (MS) [18] to be used as alternative methods, including HPLC-MS/MS [19], time of flight mass detector (HPLC-TOF) [20,21] and HPLC-Orbitrap MS [22,23,24,25]. Orbitrap MS technique is the most sensitive of these previously proposed methods [24].

The isolation and preparation of AFs were first reported by Sargeant et al. in 1961 [26]. Toxic extracts of Brazilian groundnuts were purified via column chromatography on alumina, yielding a nearly colorless crystalline material. This material was first referred to as AFs G and B by Nesbitt et al. in 1962, who purified the hot methanol extract of *A. flavus* on silica gel and further purified the AF mixture with a counter-current distribution to separate B and G groups of AFs [27]. One year later, the four main AFs (AFG_1_, AFG_2_, AFB_1_ and AFB_2_) were described for the first time and separated on silica gel with purities above 90%; moreover, the unsaturation of the furan ring in AFG_1_ and AFB_1_ and the saturation of the same ring in AFG_2_ and AFB_2_ were reported [28]. Later, Stubblefield et al. separated the crude mixture of AFs extracted from wheat and rice using a process of successive separations with a silica gel mesh as the stationary phase and chloroform/ethanol (99:1, v/v%), and chloroform/acetone/ethanol (97.25:2:0.75, v/v/v%) as eluents; the purity of each obtained AF was above 90% [29]. De Jesus et al. successfully purified three of the four AFs (AFB_1_, AFB_2_ and AFG_1_) from the solid culture of *A. flavus* extracted from both the media and the mycelia using consecutive normal-phase chromatography steps with different ratios of chloroform and acetone as the eluents; 1.09 g of AFB_1_, 360 mg of AFB_2_ and 2.1 of AFG_1_ were obtained from 21 g of crude broth and 9.7 g of crude mycelial extract [30].

Centrifugal partition chromatography (CPC) is an extensively studied technique but it is less well-known and not as commonly used as solid–liquid chromatography. In CPC, a solvent from a biphasic solvent system is immobilised by a centrifugal force in a stacked and interconnected rotating-discs (the stationary phase), while another phase (the mobile phase) is pumped through this channel and the components injected in a mixture into the mobile phase are eluted from the column according to their partition coefficients [31]. However, for the successful CPC separation, firstly the biphasic solvent system should be selected such that the sample components have different partition coefficients in a range of 0.5–2.0 in each phase [32]. CPC further allows for rapid and inexpensive method development, higher throughput, higher yields and reduced costs compared to typical preparative HPLC techniques [33]. Based on these unique advantages and the high resolution offered by CPC, this technique has been applied for the separation of numerous bioactive natural products including sesamin, catharanthine and vindoline [34,35,36,37,38] as well as certain mycotoxins [39,40,41,42,43]. For example, fumonisin B_1_ was separated from the crude extract of *Fusarium verticillioides* by CPC in a single 70-min run with a purity above 98% and total yield of 68% [39]. B-type fumonisins have also been purified by several consecutive separation steps with pentane/tert-butyl methyl ether/butan-1-ol/ethanol/1% formic acid in water and pentane/butan-1-ol/ethanol/1% formic acid in water as the solvent systems; as a result, a total of 500 mg of fumonisin B_1_, B_2_ and B_3_ was obtained with a purity above 98% from 1 kg of maize culture [40]. In another study, nivalenol and fusarenon-X were purified by passing a crude acetonitrile extract from *Fusarium graminearum* through silica gel then running two consecutive CPC separations: the first with water as the stationary phase and butanol as the mobile phase and the second with a chloroform/methanol/water (65:65:40, v/v/v%) solvent system; from 1 kg of pressed barley culture, 340 mg nivalenol and 600 mg fusarenon-X were obtained with yields of 44% and 68%, respectively [41]. Another liquid–liquid chromatographic separation with a 1:1 ethyl acetate/water solvent system (by volume) was used to purify deoxynivalenol from a rice culture of *F. graminearum*, resulting in 95% purity after several consecutive runs [42]. Similarly, a 7.5:2.5:10 hexane/ethyl acetate/pH 4 acetic acid solution (by volume) was used as the solvent system to purify the mycotoxin patulin produced by *Penicillium expansum* that was cultivated in sterilised apple juice and apple cider; the compound was purified with a maximum purity of 98.6% and a recovery of 86.2% [43].

In the present study, for the first time, CPC is applied to isolate all four AFs and completely separate them from impurities following extraction and liquid–liquid chromatographic separation procedures.

## 2. Results and Discussion

### 2.1. Cultivation and Extraction

**Figure 2:**
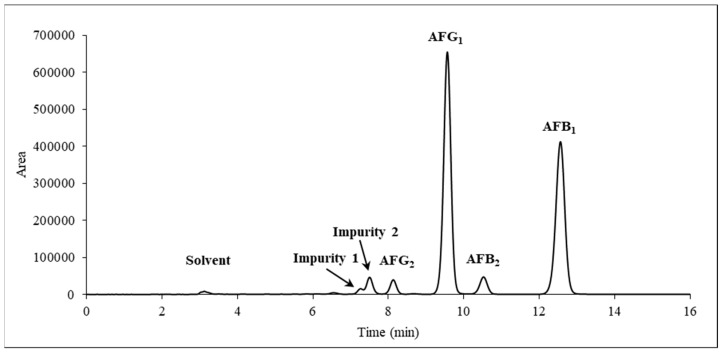
Chromatogram of the crude extract measured with high performance liquid chromatography coupled with an ultraviolet detector (HPLC-UV) at λ = 365 nm.

*A. parasiticus* SZMC 2473 strain, an ex-type strain of the species [44], was selected from Szeged Microbiology Collection (SZMC) for cultivation because it has been reported to produce both AFGs and AFBs in relatively high amounts [45]. After seven days of cultivation, the AFs were extracted in a three-step process using first dichloromethane was used, followed by a hexane/methanol/water ternary system [46] to remove the fat and other non-polar components produced by the fungus. At this stage AFs were distributed into the aqueous phase, which was partitioned again with dichloromethane. An HPLC-UV analysis (Figure 2) revealed that the *A. parasiticus* produced AFG_1_ (47.5%, R*_t_* = 9.564 min) and AFB_1_ (42.6%, R*_t_* = 12.560 min) as the major products and AFG_2_ (3.0%, R*_t_* = 8.109 min) and AFB_2_ (4.2%, R*_t_* = 10.523 min) in smaller quantities; the total impurity content was 2.7% (R*_t_* = 7.255 min and R*_t_* = 7.493 min).

### 2.2. Selection of Solvent Systems

For the separation of AFs by liquid–liquid chromatography, several ternary systems, each comprising a “bridge” solvent was used in combination with more polar and less polar partitioning solvents, were created in accordance with the “best solvent” approach [47]. Here, one protic and two aprotic solvents were selected as the best solvents. To form a non-aqueous ternary system, chloroform was used as the best solvent and was paired with hexane and acetonitrile; to form an aqueous ternary system, acetone was used with either hexane and heptane or toluene and water (Table 1). Furthermore, the protic best solvent was acetic acid, which was used in combination with diethyl ether, chloroform or toluene as the non-polar solvent and water as the polar solvent to form a ternary system (Table 2). Thus, a total of 63 biphasic systems based on these seven compositions were investigated.

#### 2.2.1. Chloroform and Acetone as Best Solvents

The results with the ternary systems containing chloroform as the best solvent showed that the P value was below 0.1 in most systems, indicating that the examined components remained mainly in the lower, chloroform-rich phase (Table 1). The two-phase region in the ternary diagram shows that it is existed only in a narrow range of chloroform contents (1–12%); therefore, these tested ternary systems covered almost the entire two-phase region of the system [31]. Hence, it can be concluded that none of the examined components could have been transferred to the upper phase; therefore, this ternary system cannot be applied for the CPC separation of AFs.

The application of the next set of ternary systems based on acetone led to similar observations. In the systems containing hexane and heptane, most of *p*-values were below 0.1, indicating that the components remained in the aqueous (lower) phase (Table 1). Examining the hexane/acetone/water ternary systems, in certain cases, the *p*-values were closer to one, indicating the successful partitioning of the AFs.

Because none of the previous two ternary systems based on chloroform or acetone fulfilled to the requirements of the CPC application, an organic phase with higher polarity was needed, leading to the use of toluene. Using toluene as the third solvent the *p*-values were higher than one (Table 1), indicating that the AFs were mainly contained in the upper phase. In the biphasic systems containing more water than toluene, the AFs and the impurities were all transferred completely into the upper phase (Table 1). Furthermore, in the ternary system containing around 65% acetone, the *p*-values varied within narrow range around one and stagnated around it (Table 1); this was likely due to the shallower slopes of the tie lines in this ternary system [31]. When the amount of toluene was 70%, the AFs are concentrated mostly in the upper phase and the *p*-values were within the acceptable range (0.9–9.9).

**Figure 3:**
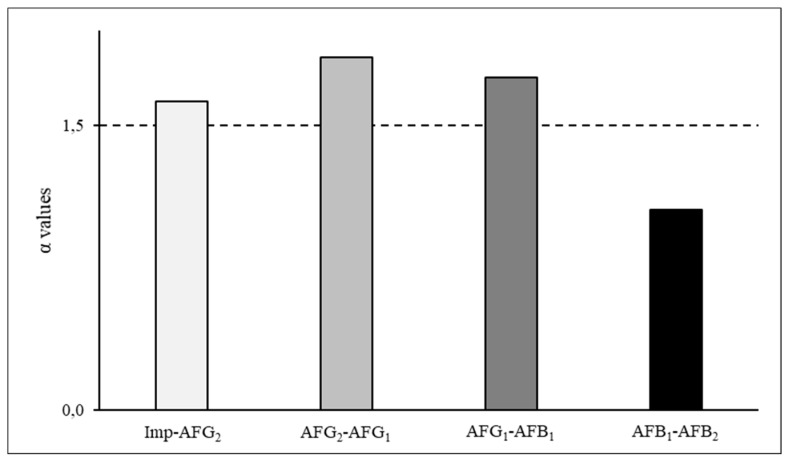
Separation factors of the components partitioned in the ternary system of 70:10:20 toluene/acetone/water. Imp: impurity; AFG_1_, AFG_2_, AFB_1_ and AFB_2_: Aflatoxins G_1_, G_2_, B_1_ and B_2_.

Furthermore, based on the quotients (α values) of the ordered *p*-values, the system composed of 70:10:20 toluene/acetone/water was able to separate the impurities from the AFs. Furthermore, using this system, AFG_1_ and AFG_2_ could be purified separately although the coelution of AFB_1_ and AFB_2_ could be expected during the CPC separation due to low α value (1.05) between these components (Figure 3).

#### 2.2.2. Acetic Acid as Best Solvent

Acetic acid was also tested as the best solvent because it has been shown to dissolve the components very well [48,49]. In addition to the acetic acid and water, diethyl ether, chloroform and toluene were tested as non-polar components (Table 2).

Of the diethyl ether/acetic acid/water systems, those with compositions of 30:10:60 and 45:15:40 were associated with low *p*-values for the AFs. The P value increased when the diethyl ether/water ratio was increased (e.g. to diethyl ether contents of 60% and 75%), but the calculated α values remained below 1.5. The above mentioned four tested compositions covered the full range of acetic acid percentages of the biphasic part of ternary diagram and due to the inappropriate partitions of the components obtained with diethyl ether, the non-polar component of the system was changed to chloroform. In these systems, the partition of the components shifted toward the lower (non-polar) phase with low *p*-values with low acetic acid concentrations (Table 2). As the tie lines of these ternary systems lean to the left, increasing the volumetric ratio of acetic acid is expected to cause its concentration in the polar phase to increase proportionally. Therefore, the acetic acid concentration was increased, causing the components to shift into the upper (polar) phase (Table 2) while maintaining α values under the acceptable limit for most component pairs. With the system containing 45% acetic acid, the components should theoretically (based on α values) be eluted in three groups: AFB_2_, AFG_1_+AFG_2_ and Imp1+Imp2+AFB_1_.

**Figure 4:**
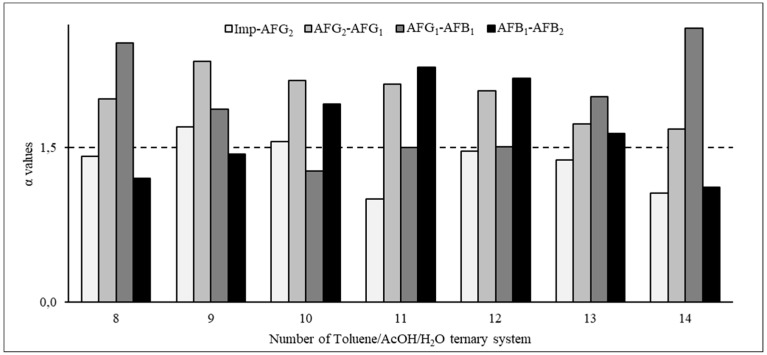
Separation factors in the toluene/acetic acid/water ternary system.

When the non-polar solvent was changed to toluene, the ratio of ascending *p*-values changed significantly (Table 2), indicating that the components tended to remain in the upper phase in these systems. The α values corresponding to these systems are shown in Figure 4. Based on these calculations, the system of 20:10:70 toluene/acetic acid/water (Table 2, system 9) could separate the AFs and the impurities but the range of the *p*-values was remarkably wide, necessitating a very long chromatographic run. When the toluene/acetic acid/water ratios were 20:20:60 (Table 2, system 11) and 20:30:50 (Table 2, system 13), the α values between the impurities and AFG_2_ were low; similarly, when this ratio was 40:30:30 (Table 2, system 14), the α value between AFB_1_ and AFB_2_ was not high enough (Table 2, Figure 4). However, when the toluene/acetic acid/water ratio was 30:24:50 (Table 2, system 12), the *p*-values were in the most suitable range (0.04 to 1.82), allowing for a CPC run that can be completed within an acceptable time and the α values were appropriate (above 1.5 for all AFs), indicating that perfect resolution can be expected (Table 2, Figure 4). Therefore, this system was selected as the optimal system for the final liquid–liquid chromatographic separation.

### 2.3. Optimisation of the CPC Method

To determine the mode (ascendant or descendant) for the liquid–liquid separation, the elution volume (*Ve*) was calculated in each mode while using the optimal theoretical mobile phase/stationary phase ratio (20:80 by volume). The retention volumes for AFB_1_, AFB_2_, AFG_1_ and AFG_2_ were predicted as 159, 304, 426 and 842 mL, respectively in ascendant mode and 1605, 1045, 560 and 243 mL, respectively, in descendant mode. Hence, the lower phase was used as the stationary phase and the upper phase was used as the mobile phase in subsequent experiments, leading to a normal-phase-like separation.

To set up the instrument for the separation, the column was filled with the stationary phase at a rotor speed of 500 rpm and flow rate of 50 mL/min. Then, the mobile phase was pumped through the system at a flow rate of 10 mL/min with an initial rotor speed of 2200 rpm, which was decreased gradually according to the observed pressure, which should be below 100 bar. Only 10 mL of the stationary phase was extruded (4:96 by volume) when the rotor speed was decreased to 2000 rpm and approximately 25 mL was extruded after reaching a stable pressure when the rotor speed was decreased further to 1800 rpm. Hence, these parameters were subsequently used for the first chromatographic run.

**Figure 5:**
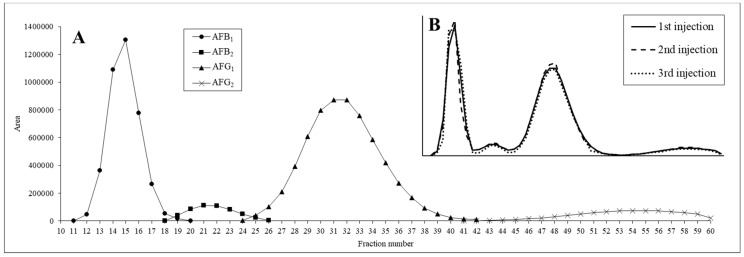
Fractograms of (**A**) the optimized centrifugal partition chromatographic (CPC) run and (**B**) repeated CPC runs.

However, this separation process proved to be considerably long due to the high retention of AFG_2_, which was only eluted from the column after 120 min. To shorten the retention time of this slowly eluted component, the flow rate was increased to 15 mL/min in the final method. The pressure of this system was only 46 bar, which is far below the maximum limit (100 bar) and the extruded volume of the stationary phase increased to 60 mL (24 v%). 90 mg of same dried AF extract, as used for the solvent system selection, was dissolved in 4 mL of each the upper and lower phases and injected to the instrument; the sample was dissolved well to preserve the integrity of the liquid–liquid system. The pressure was stable throughout the run, indicating the stability of the selected biphasic system. Using this method, the four AFs and the impurities were separated successfully: the AFs were eluted completely from the column while the impurities were retained in the system during the 75 min run time and, thus, were not included in the fractogram (Figure 5A).

After confirming that the separation was successful, the stability and repeatability of the system were evaluated by repeating the same separation process three times with fresh eluents for both the stationary and mobile phases and the same amount of crude AF sample (90 mg). The fractograms of the three consecutive runs (Figure 5B) shows that the composition of the collected fractions during the separation are fit to each other and the relative standard deviations of the AF contents in corresponding fractions were under 10%. Thus, it was concluded that the proposed separation process is repeatable.

**Figure 6:**
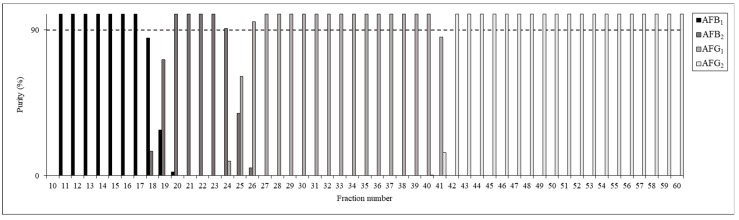
Composition and purity (%) of the fractions obtained in the optimized CPC run.

Sixty fractions were collected in total during each run, beginning immediately after the sample injection and proceeding until the 9 mL was eluted. The compositions of the fractions were evaluated by HPLC-UV (Figure 6); the results showed that most fractions had purities above 90%. Fractions 11–17 were pooled to obtain the AFB_1_ sample (24 mg), fractions 21–23 were pooled for the AFB_2_ (2 mg), fractions 27–40 were pooled for the AFG_1_ (49 mg) and fractions 44–59 were pooled for AFG_2_ (6 mg). The rest of the fractions containing mixtures of more than one AF were then pooled, neutralized and evaporated to dryness for further analyses and purification. For example, AFB_2_-rich fractions 18–20 and 24–26 contained 3 mg AFB_2_ as well as 2 mg AFB_1_ and 1 mg AFG_1_.

### 2.4. HPLC-UV and HR-MS to Verify Identity and Determine Purity

**Figure 7:**
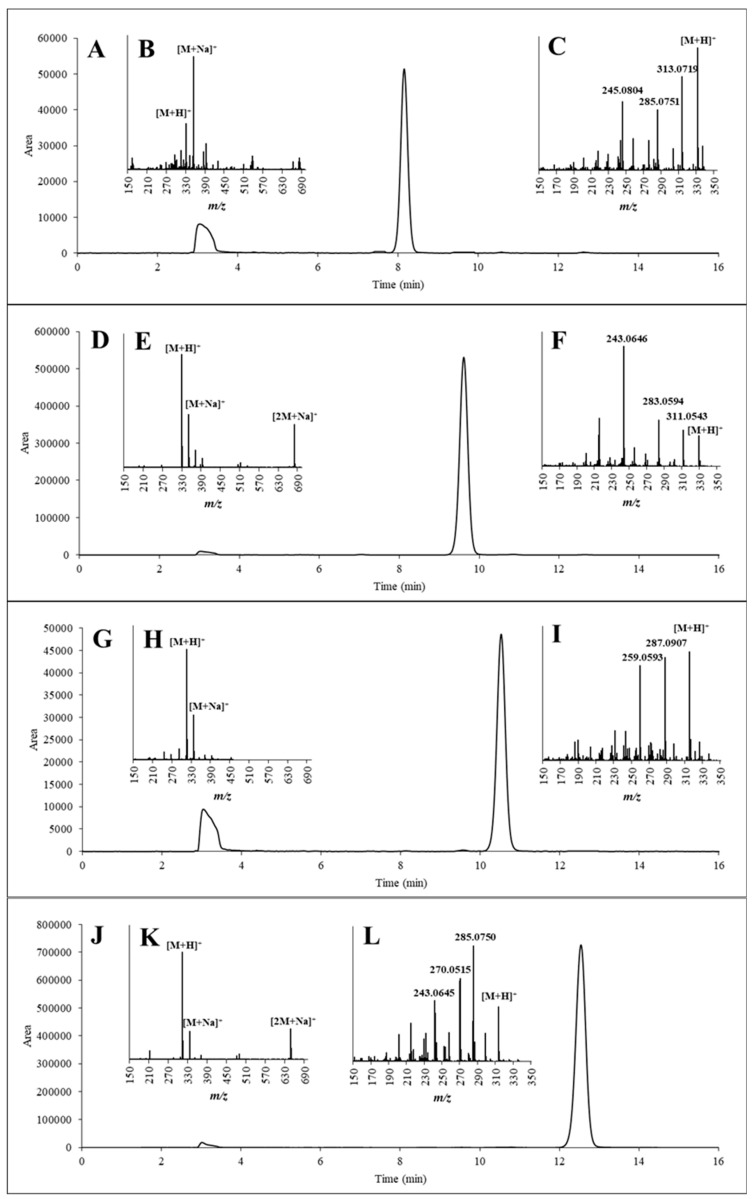
HPLC-UV chromatograms of the purified AFG_2_ (**A**), AFG_1_ (**D**), AFB_2_ (**G**) and AFB_1_ (**J**); Mass spectra of the purified AFG_2_ (**B**), AFG_1_ (**E**), AFB_2_ (**H**) and AFB_1_ (**K**); and MS^2^ spectra of the [M + H]^+^ adduct ions of the purified AFG_2_ (**C**), AFG_1_ (**F**), AFB_2_ (**I**) and AFB_1_ (**L**).

To verify the structures of the purified compounds, each sample of them was injected into an HPLC-UV and into an UHPLC-QExactive system containing both quadrupole and orbitrap analysers via flow injection. According to the HPLC-UV chromatograms (Figure 7A,D,G,J), none of the separated AFs contained major impurities and the retention times for all AFs were consistent with those of the reference standards. The adduct ions (the [M + H]^+^ and [M + Na]^+^ in all samples and [2M + Na]^+^ in AFB_1_ and AFG_1_) were detected in the full-scan measurements (Figure 7B,E,H,K). Further, after the fragmentation of [M + H]^+^ as a precursor ion, the three most intense fragment ions were selected to confirm the identities in the MS^2^ evaluations (Figure 7C,F,I,L).

The MS and MS^2^ spectra of the purified AFs were compared with those of the corresponding AF standards. The results, which are summarized in Table 3, show that the measured mass values were consistent with the *m*/*z* values of the reference compounds with a maximum deviation of only 2.55 ppm. Furthermore, the ratios between the fragment ions were only slightly different (differences were within the range of 0–5%) compared with (Table 3).

To test the purity of the separated and fractionated AFs, MS was also applied due to its high sensitivity. Hence, even considering the presence of the [M + H]^+^ molecular ions of AFs other than the purified one, the *m*/*z* values remained under 0.05% in all cases.

### 2.5. Yield of the Entire Purification Procedure

From the 4.5 L of liquid culture of *A. parasiticus* SZMC 2473 strain, a total of 1351 mg of AFs was obtained by the proposed CPC method including 442 mg of AFB_1_, 43 mg of AFB_2_, 817 mg of AFG_1_ and 100 mg of AFG_2_ with purities of 98.2%, 96.3%, 98.1% and 97.0%, respectively (Table 4). At these yields, the recovery rates from the entire procedure were 90.5%, 85.3%, 98.7% and 96.0% for AFB_1_, AFB_2_, AFG_1_ and AFG_2_, respectively, based on the original concentrations in the crude extracts as measured by HPLC-UV. Furthermore, the overall AF recovery rate was 92.6% (Table 4), indicating that the applied extraction steps did not cause any remarkable loss of AFs.

## 3. Conclusions

In this study, we developed an effective liquid–liquid chromatography method to separate the four AFs (B_1_, B_2_, G_1_ and G_2_) produced by a filamentous fungus, *A. parasiticus*. After evaluating many systems, the ternary system of 30:24:50 toluene/acetic acid/water was shown to provide an ideal range of *p*-values and an acceptable resolution for instrumental applications. Using this biphasic system, successful separation was achieved by injecting 90 mg of crude extract onto a 250 mL CPC column. Hence, approximately 1.5 g crude AF mixture could be extracted from 4.5 L ferment broth in one 75 min CPC run, yielding a total of 90 mg of crude extract. The total AF yield of the whole procedure was 92% based on the initial total AF concentration in the crude extract. The resulting recovery rates and purities were found to be sufficiently high to allow for cost-effective reference standard preparations, which is essential for the precise analytical measurements of food and feed products and keeping their mycotoxin contents within tolerable ranges.

## 4. Materials and Methods

### 4.1. Chemicals and Solvents

The following microbiological media components were used: Bacto malt extract was purchased from Becton Dickinson (Környe, Hungary) and yeast extract, NaCl and sucrose were purchased from Molar Chemicals (Halásztelek, Hungary). All solvents used for extraction were purchased from Molar Chemicals Hungary and were of analytical grade. The solvents used for the various solvent systems and HPLC-UV measurements were of gradient grade or super gradient grade and were purchased from VWR International (Debrecen, Hungary). The AF standard mixture (including AFB_1_, AFG_1_, AFB_2_ and AFG_2_) was purchased from Sigma-Aldrich (Budapest, Hungary).

### 4.2. The Producer Strain and Culture Conditions

*Aspergillus parasiticus* strain SZMC 2473 (CBS 260.67; GenBank Accession number for ITS: MG662400) was originally isolated in Japan and is the ex-type of the species [44]. To cultivate the strain, 500 mL liquid medium was prepared in 1.2 L Roux-flasks (30 g Bacto malt extract, 20 g yeast extract, 200 g sucrose in 1 L distilled water). The flasks were capped with a cotton cork and sterilised in an autoclave for 30 min at 115 °C. Each flask was inoculated with a 5 mL conidial suspension prepared in a sterilised saline solution and incubated in a horizontal position in the dark at 25 °C for 7 days.

### 4.3. Aflatoxin Extraction

AFs were extracted from fermented broth in four steps: First, extraction was performed on one litre of broth with 500 mL followed by 250 mL of dichloromethane. The organic phases were combined and evaporated to water; the volume of aqueous residue was then measured. Next, methanol and hexane were added such that the water/methanol/hexane ratio was 45:50:120 (by volume). After the phases were separated, the upper phase was removed and further extraction was performed on that phase with water/methanol (45:50 by volume). The separated water/methanol phases were then combined and dichloromethane was added until two phases formed and extracted in two repetitions. The combined organic phases were dried over MgSO_4_, membrane filtered and evaporated to dryness.

### 4.4. Testing of the Solvent Systems

The dried crude extract was dissolved in dichloromethane at a concentration of 1.8 mg/mL and split into 1-mL aliquots; the dichloromethane was then evaporated. The constituents of the solvent systems to be tested (Table 1 and Table 2) were mixed in tubes and vortexed for 10 s. When the phases were let to separate and 500 µL from both phases was added to the previously aliquoted and dried crude extract. After the phase-separation 300 µL of each phase was transferred into new vials and evaporated to dryness and subsequently dissolved in 500 µL of acetic acid prior to HPLC injection.

### 4.5. HPLC-UV Measurements

Analyses were performed using a Shimadzu HPLC system (Shimadzu, Kyoto, Japan) equipped with a DGU-14A degasser, an LC-20AD binary pump, a SIL-20A autosampler, a CTO-10ASvp column thermostat, an SPD-10Avp UV-VIS detector and a CBM-20A system controller. Class VP ver. 6.2 software was used for data acquisition and evaluation. The separation of AFs was performed on an injected sample volume of 5 µL for 16 min in a Phenomenex Gemini C18, 250 mm × 4.6 mm, 5-µm column (Phenomenex, California, USA) with the mobile phase comprising water (A) and a 1:1 mixture of methanol and acetonitrile (B) combined in an A/B ratio of 60:40 by volume. The flow rate was 1 mL/min and the column temperature was maintained at 40 °C. The peaks of the AFs and the impurities were detected at λ = 365 nm. This method was also applied for the purity determination of the fractions and the final products.

### 4.6. Evaluation of the Separation

The partition coefficient (P) of each compound was calculated by dividing the area under the peaks detected in the upper and lower phases. The separation factor (α) for each solvent system was calculated by dividing the greatest P value by the smallest one arranged in the ascending series. The elution volume (*V_e_*) was calculated as follows: *V_e_* = *V_S_* + *P*(*V_C_* − *V_S_*), where *V_S_* is the volume of the stationary phase and *V_C_* is the volume of the column.

### 4.7. Centrifugal Partition Chromatography

Preparative-scale separations were performed using a laboratory-scale 250 mL CPC column (Gilson, Saint-Ave, France) coupled with a PLC 2250 flash/prep hybrid instrument (Gilson, Saint-Ave, France) containing an UV/Vis detector, fraction collector, electronically actuated injector valve with a 10 mL sample loop and an electronically actuated four-way two-position ascendant/descendant valve. Gilson Glider Prep (Ver. 5.1) software (Gilson, Saint-Ave, France) was used to control the instrument and acquire data.

Before the instrumental analysis, the selected system was prepared in a 5 L separation funnel and shaken to saturate the phases. The phases were then separated and transferred into glass bottles. At the beginning of the instrumental analysis, the column was filled with the stationary phase. Then the mobile phase was pumped with decreased flow through the column until the system reached to hydrodynamic equilibrium. The volume of the extruded stationary phase was measured using a graduated cylinder and the ratio between the volumes of phases was calculated.

The final optimised chromatographic run was carried out in ascendant mode at 1800 rpm with a flow rate of 15 mL/min for the biphasic system comprising toluene/acetic acid/water in a volumetric ratio of 30:24:50. The separation was carried out for 75 min and 20 mL fractions were collected during the whole run, while the UV detector was set to 366 nm. The pH of each fraction fractions was neutralised by adding a saturated NaHCO_3_ solution. The AF content in each fraction was measured by HPLC-UV. The fractions containing pure AFs (> 95%) were pooled and the solvent was evaporated. The resulting white powder containing the four AFs was used for subsequent analyses.

### 4.8. HR-MS Analyses

The identities of the purified AFs were analysed using a Dionex Ultimate 3000 UHPLC system (Thermo Fischer Scientific, Waltham, USA) coupled with a Q-Exacitve Focus Orbitrap mass spectrometer (Thermo Fischer Scientific, Waltham, Massachusetts, USA) using flow injection with an isocratic eluent (20:80 water/methanol mixture by volume with 0.1% acetic acid). The capillary temperature of the heated electrospray interface (HESI) and the heater temperature were set to 250 °C. The sheath gas flow rate was 30 a.u. and the auxiliary gas flow rate was 15 a.u. The capillary voltage was set to 4.5 kV. To achieve the fragmentation of the examined molecules, the HESI capillary and the auxiliary gas were heated to 350 °C, the normalised collision energy in the collision cell of the instrument was set to 70 a.u. and N_2_ was used as the collision gas. From the purified AF solution solved in acetic acid (100 µg/mL), 5 µL was injected for analysis.

## Figures and Tables

**Table 1:** Tested ternary systems and the corresponding *p*-values.

	Solvent System	Volume Ratio	P_Imp1_	P_Imp2_	P_AFG2_	P_AFG1_	P_AFB2_	P_AFB1_
1	hexane/chloroform/acetonitrile	55:5.5:39.5	<0.10	<0.10	<0.10	<0.10	<0.10	<0.10
2		55:3.8:41.2	0.15	0.13	0.14	0.15	0.14	0.15
3		77.7:3.2:19.1	<0.10	<0.10	<0.10	<0.10	<0.10	<0.10
4		77.7:5:17.3	0.19	<0.10	<0.10	<0.10	<0.10	<0.10
5		42:9:49	<0.10	<0.10	<0.10	<0.10	<0.10	<0.10
6		34:8:58	<0.10	<0.10	<0.10	<0.10	<0.10	<0.10
7		55:7:38	<0.10	<0.10	<0.10	<0.10	<0.10	<0.10
8		63:10:27	<0.10	<0.10	<0.10	<0.10	0.11	<0.10
9		62:9:29	<0.10	<0.10	<0.10	<0.10	<0.10	<0.10
10	hexane/acetone/water	36:39:25	0.14	<0.10	<0.10	<0.10	0.14	<0.10
11		10:50:40	<0.10	<0.10	<0.10	<0.10	<0.10	<0.10
12		9:39:52	<0.10	<0.10	<0.10	<0.10	<0.10	<0.10
13		15:60:25	0.14	0.13	0.19	0.24	0.26	0.29
14		56:24:20	<0.10	<0.10	<0.10	<0.10	<0.10	<0.10
15		66:24:10	<0.10	<0.10	<0.10	<0.10	<0.10	<0.10
16		50:40:10	<0.10	<0.10	<0.10	<0.10	<0.10	<0.10
17		30:60:10	<0.10	<0.10	<0.10	0.11	0.13	0.15
18		40:50:10	0.16	0.14	0.23	0.24	0.29	0.32
19		32:63:5	0.13	0.13	0.19	0.21	0.24	0.26
20		23:77:10	0.53	0.61	0.62	0.64	0.66	0.68
21		20:70:10	<0.10	<0.10	0.14	0.14	0.18	0.20
22	heptane/acetone/water	44:29:27	0.17	0.15	0.16	0.19	0.18	0.20
23		8:65:27	<0.10	<0.10	<0.10	<0.10	<0.10	<0.10
24		27:57:16	<0.10	<0.10	<0.10	<0.10	<0.10	<0.10
25		30:60:10	<0.10	<0.10	<0.10	<0.10	<0.10	<0.10
26		40:20:40	<0.10	<0.10	<0.10	<0.10	<0.10	<0.10
27		20:10:70 ^a^	-	-	-	-	-	-
28		55:25:20	<0.10	<0.10	<0.10	<0.10	<0.10	<0.10
29		80.5:9.5:10	<0.10	<0.10	<0.10	<0.10	<0.10	<0.10
30		23:57:20	<0.10	<0.10	<0.10	<0.10	<0.10	<0.10
31		22:38:40	<0.10	<0.10	<0.10	<0.10	<0.10	<0.10
32	toluene/acetone/water	39:26:35	1.81	2.98	5.77	9.80	11.28	22.67
33		14:26:60	2.16	3.42	6.26	9.11	11.58	18.53
34		10:40:50	8.33	38.64	6.46	4.11	2.66	58.49
35		14:51:35	6.59	3.26	2.80	3.58	3.39	5.14
36		29:55:16	1.39	1.83	2.86	3.86	5.00	5.03
37		30:10:60	0.87	2.04	4.21	9.03	22.75	19.06
38		6:74:20 ^b^	-	-	-	-	-	-
39		18:12:70	0.86	1.72	2.27	6.16	13.11	12.32
40		12:67:21	1.21	1.26	1.40	1.51	1.53	1.60
41		17:67:16	1.08	1.11	1.16	1.21	1.25	1.25
42		8:67:25	1.21	1.26	1.43	1.63	1.67	1.75
43		70:10:20	0.99	1.78	2.90	5.38	9.92	9.42
44		35:55:10	1.36	1.64	2.46	3.24	4.13	4.00
45		15:55:30	1.38	1.58	2.19	2.83	2.84	3.36
46		6:55:39	1.40	1.62	2.20	2.84	3.02	3.41

^a^ Could not dissolve the sample as it was too polar. ^b^ Did not form a biphasic system.

**Table 2:** Tested ternary systems with acetic acid as the best solvent and the corresponding *p*-values.

	Solvent System	Volume Ratio	P_Imp1_	P_Imp2_	P_AFG2_	P_AFG1_	P_AFB2_	P_AFB1_
1	diethyl ether/acetic acid/water	75:5:20	0.70	0.86	1.67	1.75	3.40	3.43
2		60:20:20	0.26	0.32	0.46	0.53	0.66	4.00
3		30:10:60	0.10	0.11	0.21	0.22	0.25	0.43
4		45:15:40	<0.10	0.15	0.19	0.20	0.33	0.41
5	chloroform/acetic acid/water	26:24:50	0.42	0.37	<0.10	<0.10	<0.10	<0.10
6		36:34:30	0.47	0.93	0.18	0.16	0.11	<0.10
7		35:45:20	1.03	1.15	0.58	0.57	0.29	0.84
8	toluene/acetic acid/water	80:6:14	<0.10	<0.10	0.31	0.62	1.56	1.88
9		20:10:70	0.14	0.43	0.73	1.70	3.18	4.57
10		30:10:60	<0.10	0.4	0.63	1.36	1.73	3.33
11		20:20:60	<0.10	0.3	0.3	0.63	0.94	2.65
**12**		30:24:50	0.04	0.14	0.18	0.36	0.54	1.21
13		20:30:50	<0.10	0.12	0.16	0.28	0.56	0.92
14		40:30:30	<0.10	<0.10	<0.10	0.16	0.42	0.47
15		63:30:7	<0.10	<0.10	<0.10	0.10	0.23	0.20
16		40:42:18	<0.10	0.11	0.11	<0.10	0.23	0.22
17		20:55:25	<0.10	<0.10	<0.10	<0.10	0.31	0.21

**Table 3:** Comparisons of the masses and intensity ratios of the detected ion forms with those of the reference standards by via full-scan and MS^2^ high-resolution mass spectrometry to confirm the identities of the AFs.

	AFB_1_			AFB_2_			AFG_1_			AFG_2_		
	^c^ Ref. (*m*/*z*)	^d^ Mass dev. (ppm)	^e^ Ratio dev. (%)	^c^ Ref. (*m*/*z*)	^d^ Mass dev. (ppm)	^e^ Ratio dev. (%)	^c^ Ref. (*m*/*z*)	^d^ Mass dev. (ppm)	^e^ Ratio dev. (%)	^c^ Ref. (*m*/*z*)	^d^ Mass dev. (ppm)	^e^ Ratio dev. (%)
^a^ Full scan	313.0699	1.25	0	315.0849	0.99	0	329.0646	1.43	0	331.0811	1.17	0
	335.0504	1.56	0	337.0669	1.18	0	351.0464	1.26	0	353.0631	1.66	0
	647.1142	2.02	0	-	-	-	679.1039	2.54	0	-	-	-
^b^ MS^2^	285.0750	1.99	5	287.0907	1.37	1	311.0543	2.57	1	313.0719	2.03	0
	270.0515	1.04	3	259.0593	1.18	2	283.0594	1.79	3	285.0751	1.98	1
	243.0645	2.30	1	-	-	-	243.0646	2.01	4	245.0804	2.13	1

^a^ The full-scan *m*/*z* values are consistent with the molecular ion adducts including [M + H]^+^, [M + Na]^+^ and [2M + Na]^+^. ^b^ The presented product ions originated from the fragmentation of the protonated molecular ions. ^c^ The *m*/*z* values of the reference standards. ^d^ Mass deviation of the purified compounds from the corresponding ion reference standards determined with the high-resolution mass spectrometer. ^e^ Differences between the ratios of the formed ion intensities measured in the reference standards and the purified components.

**Table 4:** Purification efficiencies of the AFs from *A. parasiticus* liquid culture in each separation step.

		AFB_1_	AFB_2_	AFG_1_	AFG_2_	Total AFs
Crude extract (culture medium → dichloromethane)	Yield (mg)	442	40	827	105	1414
	Purity (%)	39	4.0	45.4	2.9	91.3
Second extract (dichloromethane → hexane/methanol/water)	Yield (mg)	442	40	827	105	1414
	Purity (%)	41.7	4.0	46.9	3.0	95.6
	Recovery (%)	100	100	100	100	100
Third extract (hexane/methanol/water → dichloromethane)	Yield (mg)	442	40	827	105	14141
	Purity (%)	42.6	4.2	47.5	3.0	97.3
	Recovery (%)	100	100	100	100	100
CPC separation (final product) (30:24:50 toluene/acetic acid/water)	Yield (mg)	400	34	817	100	1351
	Purity (%)	**98.2**	**96.3**	**98.1**	**97.0**	**97.3**
	Recovery (%)	90.5	85.3	98.7	96.0	92.6
Whole procedure	Recovery (%)	90.5	85.3	98.7	96.0	**92.6**

Note: AFB_1_, AFB_2_, AFG_1_ and AFG_2_: aflatoxins B_1_, B_2_, G_1_ and G_2_; Total AF: sum of the amounts of the four detected AFs; CPC: centrifugal partition chromatography.
